# Supplementary material for: A set of RT-PCR assays for detection of all known avian paramyxoviruses and application in surveillance of avian paramyxoviruses in China
Source: PeerJ. 2021 Mar 4;9:e10748. doi: 10.7717/peerj.10748 (PMC7937338; doi:10.7717/peerj.10748)
Supplement: Supplemental Information 2 [file peerj-09-10748-s002.doc]

**Supplementary Table 1**

The sequences of the 20 dsRNA positive control standards.

| APMVs | Nucleotide sequence^a^ | Target primer |
| --- | --- | --- |
| APMV-1 | **AAGTACTGTCTTAATTGGAGATA**TCAGACAATCAAACTGTTCGCTCATGCCATCAATCAGTTGATGGGCCTACCTCACTTCTTTGAGTGGATTCACCTAAGACTGATGGACACTACAATGTTCGTAGGAGACCCTTTCAATCCTCCAAGTGACCCTACTGACTGTGACCTCTCAAGAGTCCCTAATGATGACATATATATTGTCAGTGCCAGAGGGGGTATCGAAGGATTATGTCAGAAGCTATGGACAATGATCTCTATTGCTGCAATCCAACTTGCTGCAGCTCTAATGATGACATATATATTGTCAGTGCCAGAGGGGGTATCGAAGGATTATGTCAGAAGCTATGGACAATGATCTCTATTGCTGCAATAGATCGCATTGTCGCGTTGCCTGT**ATGGTACAGGGTGATAATCAAGT** | 1/3F, 1R |
| APMV-2 | **AACGGTGTGTCAATGGAACA**ACTGTCTTTGACAAAGAGCTTGCTTGCAATGTCACAGTTAGCACCCAGGATATCTTCAGTTCGCCAGGCGACAGCACGTAGACAGGACCCAGGACTCAGCCACTCTAATGGTTGTAATCACATTGTAGGAGACTTAGGCCCACACCAGCAGGACAGACCGGCCCGGAAGAGTGTAGTCGCAACCTTCCTTACAACAGATCTTCAAAAATATTGCTTGAATTGGCGATATGGGAGTATCAAGCTTTTCGCCCAAGCCTTAAACCAGCTATTCGGAATCGAGCATGGGTTTGAATGGATACACCTGAGACTGATGAATAGCACCCTGTTTGTCGGGATTGAAGGGTTATGCCAGAAGCTG**TGGACCATGATTTCAATAAGCA** | 4F, 4R |
| APMV-3 | **GTATCTTACTCCCTCAAAGAGAA**GGAGGTAAAGACAGATGGTCGAATCTTCGCTAAGCTCACAAGGAAAATGCGGAGCTGTCAGGTGATATTCGAAGAATTGCTAGCAGAGCATGTCTCTCCATTATTTAAAGACAACGGCGTTACTATGGCCGAATTGTCACTTACTAAGAGCCTACTCGCTATTAGCAACCTAAGCTCCACCTTATTTGAGACGCAAACAAGGCAAGGCGATCGGAATGCAAGATTCACGCATGCGCACTTCATAACAACTGACCTGCAAAAGTACTGTTTAAACTGGAGGTACCAGAGTGTAAAACTGTTCGGATCCATTGCATACTAATGAAGTC**CACTATGTATGTCGCAGACCC** | 2F, 2R |
| APMV-4 | **GTCTCATACTCACTTAAAGAGAA**GGAAGTAAAGAAAGAAGGGCGCATTTTCGCAAAAATGTCACAAAAGATGAGAGCATGCCAGGTTATTTGTGAAGAATTGCTAGCACATCATGTGGCTCCTTTGTTTAAAGAGAATGGTGTTACTCAATCAGAGCTATCCCTGACAAAAAATTTGTTGGCTATTAGCCAACTGAGTTACAACTCGATGGCCGCTAAGGTGCGATTGCTGAGGCCAGGGGACAAGTTCACTGCTGCACACTATATGACCACAGACCTAAAGAAGTACTGTCTTAATTGGCGGCACCAGTCAGTCAAACTGTTCGGATACATGTCCGTCTCACCAACAG**CACTATGTACGTCGCTGACCC** | 2F, 2R |
| APMV-5 | **AAGTATTGCCTTAATTGGAGGTA**CCCGACTATTAAATTGTTTGCCATGAAATTAAACCAGATTCTAGGGATCCCTCATGGTTTTGAATGGATTCATCTTCGCCTCCGGGACACAACTATGTTTGTTGGGGATCCCTACAATCCGCCAAATGACATTAATTTTATCAACTTGGATGACCAAAAGAATGACAGTATTTTCATAGTTTAATGACATTAATTTTATCAACTTGGATGACCAAAAGAATGACAGTATTTTCATAGTTTCTCCTAGAGGCGGAATTGAAGGCT**TATGCCAAAAAATGTGGACTATG** | 1/3F, 3R |
| APMV-6 | **AAATATTGTCTCAATTGGAGGTA**TACTGTGGTAAAACCATTTGCTCAAAGGCTGAACCAACTGTTCGGTATTGCACATGGCTTTGAATGGATTCATCTCCGACTTATGAACACAACCATGTTTGTTGGAGATCCGCACAATGTCCCCCAGTTTTCAACTAACAAGGACCTCGATTCTCAAGAGAATGATGGAATCTTCATAGTGTCAGTTTTCAACTAACAAGGACCTCGATTCTCAAGAGAATGATGGAATCTTCATAGTGTCCCCTCGTGGAGGAATTGAAGGGT**TATGCCAAAAAATGTGGACTATG** | 1/3F, 3R |
| APMV-7 | **AAATACTGCCTCAATTGGAGATA**TGAATCAATAAAATTATTTGCAAGAGCACTTAACCAATTATTTGGAATACCCCATGGATTTGAATGGATACACTTAAGGCTCATAAGAAGTACAATGTTTGTTGGGGATCCTTACAATCCTCCTGCATCAATCCAATCTTTGGATCTCGATGAACAGCCTAATGATGATATTTTTATTGTCTGCATCAATCCAATCTTTGGATCTCGATGAACAGCCTAATGATGATATTTTTATTGTCTCGCCACGTGGTGGGATTGAAGGAT**TATGTCAGAAGATGTGGACACTC** | 1/3F, 3R |
| APMV-8 | **AACGGCGTTTCGATGGAGCA**ATTGTCATTGACCAAGAGTCTACTTGCAATGTCTCAACTCTCACCAAAAGTCTCGACTCTGCAGGACACTGCATCACGTCATGTAGGCAACTCAAAATCTCAGATCGCAACCAGCAACCCATCTCGGCATCACTCAACAACCAATCAGATGTCACTCTCAAATCGGAAAACGGTTGTAGCAACTTTCTTAACAACTGATTTGGAAAAATACTGCCTGCAGTGGCGATACTCGACTATTAAGTTGTTTGCACAAGCTCTAAATCAACTCTTTGGGATTGATCACGGATTTGAATGGATACATTTAAGACTCATGAACAGCACCTTATTTGTCGGTATAGAGGGTTTATGTCAGAAGATG**TGGACCATGATATCAATTAGTG** | 4F, 4R |
| APMV-9 | **AAGTACTGTCTCAATTGGAGGTA**TCAGACCATCAAGCCTTTTGCCCATGCGATTAATCAGCTGACAGGGCTTGATTTGTTTTTTGAGTGGATCCACCTTCGTCTAATGGATACCACTATGTTCGTTGGAGATCCATACAACCCACCCTCTGATCCAACAATTGAAAACCTGGATGATGCACCCAATGATGATATCTTTATTGTAAGCGGAAGAGGAGGGATCGAGGGATTATGTCAAAAGCTTTGGACTACCATATCAATATCCGCAATACAATTAGCAGCCACCAAACCTGGATGATGCACCCAATGATGATATCTTTATTGTAAGCGGAAGAGGAGGGATCGAGGGATTATGTCAAAAGCTTTGGACTACCCGGTCAAAGTGTAGGGTAGCCTGT**ATGGTGCAAGGTGACAATCAGGT** | 1/3F, 1R |
| APMV-10 | **AATGGTGTCTCAATGGAGCA**ATTGTCACTCACAAAAAGCCTGCTTGCAATGTCTCAGCTGTCACCAAGAGTTGCCGCCACGAAGCTAGGGCTTAAGAGAGCAACTGAAAAGGATCAAGCTGCAGTCTCAATGGATTACGGTAATCACAACATTGAACTATCCCACAAGGGAGGATTGAGGAAGAGGAAAACAGTCACTGCGGGTTTTCTAACTACTGACCTTCAAAAGTATTGCCTAAATTGGAGGTATGCCAGTATCAAGCTGTTTGCCCAAGCACTTAATCAACTATTCGGCATCTCGCATGGATTTGAATGGATTCACCTCAGACTGATGGACAGTACCCTATTTGTAGGGATTGAGGGATTGTGTCAAAAGATG**TGGACAATGATTTCAATAAGCA** | 4F, 4R |
| APMV-11 | **AACGGAGTATCCATGGAGCA**ACTAGCTCTGACAAAATCATTACTAGCAATGTCTCAACTATCTCCTAGGGTAGCTGAGCACTCACCTAGGATCA-AGCGTGAAGGTGACATTAAGAAATCAAGATTAAACATAGACAAACAATTCATCAATCACGATTCCCTAGATAGTTGTTCAAAAAATGATTACAAAAGAAATCTACACGGGATTAGAAAGGAGAAAATCACAATAGCTACTTTTTTGACCACTGATTTGCAAAAATATTGTCTCAACTGGAGGTACGAATCAATTAAATTGTTTGCTATGGCCTTGAATCAGCTATTTGGTATCCCACATGGTTACGAGTGGCATCATCTATTGAAGGATTGTGTCAAAAAATG**TGGACTATGATTTCAATTAGCG** | 4F, 4R |
| APMV-12 | **AAATATTGCCTAAATTGGAGAT**ACCAGACAATAAAACCATTCGCAAGAGCAATCAACCAGCTTATGGGTCTCGAGCACTTCTTTGAATGGATACATTTGCGCCTGATGAATACTACTATGTATGTAGGAGACCCGTACAACCCTCCTCAGGAAGTAGTGACAGGGGATATTAACGATCAGCCTAATGATGACATCTTCATAGTAAGTGCAAGAGGTGGAATAGAGGGTCTTTGCCAAAAACTCTGGAGTATGATCTCAATATCAGCTATCAACCTGGCAGCTACGTCATAGTAAGTGCAAGAGGTGGAATAGAGGGTCTTTGCCAAAAACTCTGGAGTATGATCTCAATATCAGCTATCAACCTGGCAGCTACAGAGCAGACTGCAGAGTAGCCTGC**ATGGTACAAGGGGACAACCAAGT** | 1/3F, 1R |
| APMV-13 | **AAATACTGCCTTAATTGGAGATA**TCAGACCATAAAGCCCTTTGCAAGAGCTCTGAACCAGTTGCTTGGATTTGATCATTTCTTCGAATGGATACATCTAAGGTTAATGAATACAACCATGTTTGTTGGTGATCCGTACAATCCTCCAGCTGAAATTGTGAGTGGTGACATTAATGACCAGCCAAATGACGATATATTCATAGTGAGTGCAAGGGGGGGGATTGAAGGGCTATGTCAAAAACTATGGTCTATGATCTCTATTGCCGCTATCAACCTGGCAGCAGCAACGATATATTCATAGTGAGTGCAAGGGGGGGGATTGAAGGGCTATGTCAAAAACTATGGTCTATGATCTCTATTGCCGCTATCAACCTAGATCCTCTTGCAGAGTGGCGTGC**ATGGTGCAGGGAGATAACCAGGT** | 1/3F, 1R |
| APMV-14 | **aagtactgcttaaactggagata**ccctacaatcaagttatttgcgcaaaaactaaaccaactattaggcatccctcacggttatgagtggcatcatctgagacttagggacactactatgtttgttggtgacccatcagaaccaccacaggacgtttcaagtgccagcctagatgaccaaccaaatgaagggatattcatcgtgtcaggacgtttcaagtgccagcctagatgaccaaccaaatgaagggatattcatcgtgtcccccaggggtgggattgaagggc**tatgccagaaaatgtggagtacc** | 1/3F, 3R |
| APMV-15 | **aatggcgtgagcatggagca**attatcattaactaagagtctactatctatgtctcaactagcgcctaggatctcaactctcggattacgcccaattcggaataatcgtacccgaccaaagtatgatgaaaaaaatgaaagttcagcaaatttcagtggagaccctaggggtgcattagttcgtgacaagacggttgttgcaactttcttaaccacggacttacaaaaatattgtcttaattggagatattcgtcaattaaattatttgctcaagctctcaatcaattatttggacttgatcatggattcgaatggatacacttacgtttaatggattctacgatgtttgtgggtattgaagggctgtgccaaaaacta**tggactatgatttcaattagta** | 4F, 4R |
| APMV-16 | **aaatactgtttgaactggagata**ccaaaccattaaactctttgctcatgccattaatcagttgatggggttacctcacttcttcgaatggattcatctcagattaatggatacaacaatgtttgtgggggacccttttaatccgcccagcaacccatctgacataaacctggatgatgcgcagaacgatgacatattcattgtaagtgcacgtggagggatagaaggattatgccagaagctgtggacaatgatttccatatctgctatccagcttgctgctgcattcattgtaagtgcacgtggagggatagaaggattatgccagaagctgtggacaatgatttccatatctgctatccagcttgctgctgagagcacattgtagagtcgcatgc**atggtacaaggggacaaccaggt** | 1/3F, 1R |
| APMV-17 | **aaatattgtactaattggaggta**tcaggttatcaagccatttgcaagatctctgaacaggctactaggatttgaccacttctttgaatggatacacctcaggttgatggatctgaccatgtacgtaggagacccttttaaccctcctattgacatcgtgacaggcgacatcaatgatcaacccaatgatgacattttcattgtaagcgcacgtggagggatagagggactctgtcagaaattgtggacgatgatatccatatctgctatcaacctggctgcagcctttcattgtaagcgcacgtggagggatagagggactctgtcagaaattgtggacgatgatatccatatctgctatcaacctggctgcacgagctggctgtagagtggcttgc**atggttcaaggtgacaatcaggt** | 1/3F, 1R |
| APMV-18 | **aagtattgtacaaactggagata**tcaagttattaagccattcgcaaggtccttaaaccgcctcctgggctttgaccacttcttcgagtggatccatcttcgtttagctgacctgactatgtatgtaggcgacccattcaacccccctgtggacgtagccaccggagatattaatgatcagccgaacgatgacatcttcattgttagcgctcgggggggcatagagggcctatgtcaaaaactgtggaccatgatctcaatctctgcaatcaacctggccgccgcatgacatcttcattgttagcgctcgggggggcatagagggcctatgtcaaaaactgtggaccatgatctcaatctctgcaatcaacctgcgttccaactgcagagtggcatgc**atggtgcagggtgacaaccaggt** | 1/3F, 1R |
| APMV-19 | **aaatactgcactaactggaggta**tcagtcgatcaaattgtttgcccgaacattgaatcgccttttcggattcaaccacttcttcgaatggatccatctgcggctgatgaacttgaccatgtatgttggggacccatttaaccctccgactgcctgcaatgggcctgatctcgacgatcagcagaatgaggatatctttatcatcagtgcccgagggggaattgaaggattatgccaaaaactgtggtcgatgatatccattgctgctatcaatctcgcagccacaggatatctttatcatcagtgcccgagggggaattgaaggattatgccaaaaactgtggtcgatgatatccattgctgctatcaatctcaaagcgaactgcagagtcgcatgc**atggtccagggcgacaaccaagt** | 1/3F, 1R |
| APMV-20 | **AATGGTGTATCAATGGAACA**ATTATCACTTACGAAGAGCTTATTGGCGATGTCCCAATTGGCGGTAAGAGTTTGCCCGCTGCATGATCATATTGCAACCCCATATTCCTCTTCTAAAAGTGAATCAAGCATAACTAAAGAAACCATTAAGCCAGGGTTAAAGATCCAAGAGAAATTCAGCTCTCAAAGGAAGAAAGCTGTTGTAGCCACATTCTTGACAACTGATCTTCAAAAATATTGTTTAAATTGGAGATACTCCACAATTAAGCTGTTTGCCCTAGCTCTCAACCAAATATTTGGGATTGAACATGGGTTCGAGTGGATCCACTTACGTTTGATGAATTCGACACTTTTCATTGAGGGATTGTGTCAGAAACTT**TGGACCATGATTTCTATATCAA** | 4F, 4R |

^a^The nucleotides for primer binding are indicated in bold.
